# Editorial: RNA plasticity: novel structures, shapes, modifications, and functions

**DOI:** 10.3389/fpls.2023.1265867

**Published:** 2023-09-11

**Authors:** Ulhas S. Kadam, Rupesh Deshmukh, Li Tian

**Affiliations:** ^1^ Division of Life Science and Division of Applied Life Science (BK21 Four), Plant Molecular Biology and Biotechnology Research Center, Gyeongsang National University, Jinju-si, Gyeongsangnam-do, Republic of Korea; ^2^ Department of Biotechnology, Central University of Haryana, Mahendragarh, Haryana, India; ^3^ Key Laboratory of Quality and Safety Control for Subtropical Fruit and Vegetable (Ministry of Agriculture and Rural Affairs), Collaborative Innovation Center for Efficient and Green Production of Agriculture in Mountainous Areas of Zhejiang Province, College of Horticulture Science, Zhejiang A&F University, Hangzhou, China

**Keywords:** dsRNA, RNA methylation, RNA structure, RNA silencing, RNA editing, RNA splicing

This Research Topic on “*RNA Plasticity: Novel Structures, Shapes, Modifications, and Functions*” was constituted with a focus on various aspects of plant RNA biology including novel structures, modifications, and its biological roles as regulatory molecules. In applied biotechnology, novel designs drive technological innovations and advancements that help resolve contemporary society’s significant challenges.

The diversified roles played by the different forms of RNA molecules in the past few decades exemplify its significant role in cellular regulation, as it acts as a messenger of information from genomic DNA to the production of distinct proteins as well as regulates unique biological processes via flexibility of RNA structure and functions. The diversity of design and roles of RNA types has recently increased rapidly (Anderson et al., 2018; Arribas-Hernandez and Brodersen, 2020). In addition to mRNA, the noncoding RNAs (miRNA, sRNA, siRNA, and so on) is becoming better understood and accepted as a dynamic and effective regulator of cellular activities in plants (and other organisms). RNA biology has immense potential to improve food, agriculture, and the environment through enzymatic action, different molecular interactions (RNA-protein, RNA-RNA, RNA-DNA, RNA-small molecules or ions), RNA splicing (Kadam et al, 2014; Kadam et al, 2017), chromatin remodeling, and other mechanisms can assist regulate cellular physiology and activities.

For instance, the double-stranded RNA (dsRNA) produces host-specific gene silencing in many insects; such dsRNAs are being utilized to reduce pests in agricultural and horticultural crops. Although many forest pests are susceptible to RNAi, their use in forests has gotten little attention. Before forest pest suppression, dsRNA specificity, efficacy, and behavior must be determined. In their article Bragg and Rieske investigated hydroponically applied exogenous dsRNA translocation and retention in a commercially and ecologically important hardwood tree, white oak (*Quercus alba* L.). dsRNA recovery in treated seedling material was confirmed by gel electrophoresis and Sanger sequencing. Both approaches demonstrated that white oak tissues absorbed and translocated exogenous dsRNAs. According to the findings, such root uptake of dsRNAs in hardwood seedlings may protect single trees against certain tree-feeding pests or illnesses.

RNA N6-methyladenosine (m6A) is a modification in eukaryotic mRNAs (Anderson et al, 2018). m6A was discovered in wheat over 40 years ago, but its functions remain unknown. Huang et al. report that m6As in the transcriptome of hexaploid wheat spikelets during the flowering stage. Authors discovered they are evenly distributed across the A, B, and D subgenomes, while their extents and placements fluctuate across homologous genes. Homeologous genes with similar expression levels are enriched in m6A-methylated genes. The effects of m6A methylation on mRNA translation are negatively related to mRNA expression levels. m6As in coding sequences and 3’-UTRs inhibit mRNA translation, but those at start codons and 5’-UTRs enhance it. The presence of m6A-containing mRNAs in “translation” and “RNA transport” processes and pathways suggest that they may influence the translation of genes involved in translation regulation. Small RNAs (miRNA and phasiRNA) inhibit translation more than m6A methylation, and no synergistic effect was seen. It is proposed that m6A methylation status triggers translation regulation amplification. These data suggest that m6As controls translation in hexaploid wheat.

Marin-Sanz and Barro present their investigation of the transcriptome of E82 grain and leaf tissues during grain filling in order to better understand regulatory pathways in response to gliadin silencing. Gluten proteins provide wheat dough with viscoelastic properties (Shewry and Halford, 2002) but can also cause celiac disease. To reduce wheat immunogenicity, strong gliadin-silenced RNAi wheat lines were developed. The E82 line had the greatest reduction in gluten, while other grain proteins increased, resulting in a total nitrogen concentration comparable to the wild type. Grain SSPs, -amylase/trypsin inhibitors, lipid transfer proteins, serpins, and starch synthesis are all governed by a network of putative transcription factors (TFs). The leaf of E82 had numerous differently expressed genes, which were enriched in nutrition availability and transport. Many of the genes that were down-regulated were related to protease activity, amino acid and sugar metabolism, and transport in leaf-grain source-sink communication (Marin-Sanz and Barro). Proline and lysine-histidine transporters were down- and up-regulated in the leaf. Lysine-rich globulins compensate for gliadin silencing in the RNAi line, indicating that these proteins are regulated independently of the other SSPs. These findings help to interpret the complex TF network during grain loading by explaining protein compensatory mechanisms.

Post-transcriptional gene regulation is mediated by RNA methylation. Plants contain around wide range of unique RNA modifications (Arribas-Hernandez and Brodersen, 2020). RNA methylation in animals has been studied for its biological significance and applications. The methylation of plant RNA is still unknown. As plant science research has advanced, RNA methylation has become increasingly important in plant development. In the final article of the Research Topic, Shinde et al. highlight the importance of RNA methylation in plant development in their review. Plants primarily methylate RNA with N6-methyladenosine (m6A). Mutations influence Arabidopsis phenotypic changes in writers, erasers, and RNA methylation readers. Unmethylated TRANSLATIONALLY CONTROLLED TUMOR PROTEIN 1-messenger RNA does not pass from shoot to root, whereas methylated TCTP1-mRNA does (Yang et al., 2019). Plant RNA methylation research has been transformed by methylated RNA immunoprecipitation and next-generation sequencing. This method has been used to study transcriptome-wide RNA methylation in rice, Arabidopsis, Brassica, and maize. The majority of whole transcriptome methylation RNA detection methods, such as MACS2, are model-based. Finally, the limitations and prospects of RNA methylation research have been discussed.

In summary, this Research Topic has reported several discoveries on the multifaceted nature of RNAs in plants. RNA plasticity empowers the coordination and regulation of gene expression, quality control, immune responses (biotic and abiotic pressures), growth, and development. Moreover, the increasing evidence of RNA plasticity in various forms and modifications in higher organisms indicates that the highly evolved regulatory mechanisms are established, which control and integrate with other complex processes like epigenetic modifications via RNA editing, splicing, and structure. We hope this special Research Topic will provide you with significant insights into the versatile roles of RNA in plant biology.

## Author contributions

UK: Conceptualization, Formal Analysis, Funding acquisition, Validation, Writing – original draft, Writing – review & editing. RD: Formal Analysis, Resources, Writing – review & editing. LT: Formal Analysis, Investigation, Methodology, Writing – review & editing.
